# Expression of proteins associated with adipocyte lipolysis was significantly changed in the adipose tissues of the obese spontaneously hypertensive/NDmcr-cp rat

**DOI:** 10.1186/1758-5996-6-8

**Published:** 2014-01-27

**Authors:** Jie Chang, Shinji Oikawa, Hitoshi Iwahashi, Emiko Kitagawa, Ichiro Takeuchi, Masao Yuda, Chieko Aoki, Yoshiji Yamada, Gaku Ichihara, Masashi Kato, Sahoko Ichihara

**Affiliations:** 1 Graduate School of Regional Innovation Studies, Mie University, 1577 Kurimamachiya-cho, Tsu 514-8507, Japan; 2 Department of Occupational and Environmental Health, Nagoya University Graduate School of Medicine, Nagoya, Japan; 3 Department of Molecular and Environmental Medicine, Mie University Graduate School of Medicine, Tsu, Japan; 4 Health Technology Research Center, National Institute of Advanced Industrial Science and Technology, Tsukuba, Japan; 5 Department of Engineering, Nagoya Institute of Technology, Nagoya, Japan; 6 Department of Medical Zoology, Mie University Graduate School of Medicine, Tsu, Japan; 7 Department of Human Functional Genomics, Life Science Research Center, Mie University, Tsu, Japan; 8 Present address: Graduate School of Applied Biological Sciences, Gifu University, Gifu, Japan; 9 Present address: Applied Science, Roche Diagnostics, Tokyo, Japan

**Keywords:** Metabolic syndrome, Adipose tissue, DNA microarray, Proteomics, Lipolysis

## Abstract

**Background:**

The etiology of the metabolic syndrome is complex, and is determined by the interplay of both genetic and environmental factors. The present study was designed to identify genes and proteins in the adipose tissues with altered expression in the spontaneously hypertensive/NIH –corpulent rat, SHR/NDmcr-cp (CP) and to find possible molecular targets associated with the pathogenesis or progression of obesity related to the metabolic syndrome.

**Methods:**

We extracted RNAs and proteins from the epididymal adipose tissues in CP, SHR/Lean (Lean), and Wistar Kyoto (WKY) rats and performed microarray analysis and two-dimensional difference in gel electrophoresis (2D-DIGE) linked to a matrix-assisted laser desorption ionization time-of-flight tandem mass spectrometry (MALDI-TOF/TOF MS).

**Results:**

The results showed different mRNA and protein expression levels in the adipose tissue: oligo DNA microarray identified 33 genes that were significantly (P < 0.01) up-regulated and 17 genes significantly down-regulated in CP compared with WKY and Lean rats at both 6 and 25 weeks of age. The affected genes-proteins were associated with lipolytic enzymes stimulated by peroxisome proliferator-activated receptor (PPAR) signaling. Further analysis using the 2D-DIGE connected with MALDI-TOF/TOF analysis, the expression of monoglyceride lipase (MGLL) was significantly up-regulated and that of carboxylesterase 3 (CES3) was significantly down-regulated in 6- and 25-week-old CP compared with age-matched control (WKY and Lean rats).

**Conclusions:**

Our results suggest the possible involvement of proteins associated with adipocyte lipolysis in obesity related to the metabolic syndrome.

## Introduction

Obesity is currently an important public health problem worldwide [1]. Many overweight individuals suffer various metabolic abnormalities such as dyslipidemia, hypertension, and abnormal glucose tolerance associated with insulin resistance [2,3]. Moreover, the cardiometabolic risk in overweight and obese adults need to be reduced through both clinical and public health programs [4]. Evidence suggests that excessive adipose tissue worsens morphologic and dynamic cardiac function and induces cardiac abnormalities due to neurohormonal activation and increased cytokine production [5,6].

It is difficult to study the mechanisms of the metabolic syndrome in humans due to the involvement of heterogeneous genetic background and lifestyle. The spontaneously hypertensive rat/NDmcr-cp (SHR/NDmcr-cp) is a new genetically obese strain that develops hypertension, hyperlipidemia, and insulin-independent diabetes [7]. The genetic background of SHR/NDmcr-cp includes the SHR, which carry nonsense mutation of leptin receptor, which is derived from obese Koletsky rats [8]. Obese SHR/NDmcr-cp show morbid obesity compared with the lean littermates. Since the phenotype of SHR/NDmcr-cp is similar to that seen in patients with the metabolic syndrome, SHR/NDmcr-cp are thought to be one of the most suitable animal models of the metabolic syndrome [9].

DNA microarray analysis is not only a useful strategy for the identification of functional genes in several model organisms [10], but also considered as a powerful tool to visualize the molecular mechanism underlying complex biological processes [11]. In addition, the field of proteomics provides a systematic approach for quantitative and qualitative mapping of the whole proteome [12]. Proteomics analysis is used to complete complement of proteins expressed by a biological system under different physiological or pathological conditions [13]. Given that examining changes in the proteome offers insight into cellular and molecular mechanisms that cannot always be obtained through genomic analysis, we performed DNA microarray and proteomics analyses simultaneously to investigate changes in gene and protein profiles.

The adipose tissue secretes a number of proteins, in addition to being source of energy in the body [14]. In the present study, we investigated changes in the expression of genes and proteins in adipose tissues in an animal model of the metabolic syndrome to find possible molecular targets associated with obesity related to the metabolic syndrome.

## Material and methods

### Animals

SHR/NDmcr-cp (CP), SHR/Lean (Lean), and Wistar Kyoto (WKY) rats were purchased from the Disease Model Cooperative Research Association (Kyoto, Japan) [15]. In our previous study, CP exhibited obesity, hypertension, and dyslipidaemia, including hypercholesterolaemia, at a young age and presented all the features of the metabolic syndrome such as central obesity, hyperglycaemia, hypercholesterolaemia, hypertriglyceridaemia, hypertension, and insulin resistance beyond the 24 weeks of age [16]. In the present study, we used CP at 6 weeks of age as a model for the early stage of the metabolic syndrome and at 25 weeks of age as a model for the chronic stages of the metabolic syndrome. The animals were fed normal diet and housed at a temperature-controlled (25°C) environment with a 12-hour light–dark cycle. The investigation conformed to the Guide for the Care and Use of Laboratory Animals published by the US National Institutes of Health (NIH Publication No. 85–23, revised 1996) and was approved by the Committee on Laboratory Animals Utilization of Mie University.

### Measurements of blood pressure and biochemical tests

Systolic blood pressure was measured in conscious rats by the tail-cuff method as described previously [17]. Blood was collected from anesthetized rats, transferred to a chilled tube containing heparin, and centrifuged. Plasma was stored at -80°C until analysis. Serum levels of triglyceride, total cholesterol, and glucose were outsourced to SRL (Tokyo, Japan).

### Preparation of RNA

Epididymal adipose tissue was obtained from 6 or 25 week old rats and frozen immediately in liquid nitrogen and stored at -80°C. Total RNA was extracted from about 250 mg of epididymal adipose tissue using the RNeasy Lipid Tissue Mini Kit (Qiagen, Valencia, CA), according to the instructions provided by the manufacturer. The concentration of the total RNA was quantified by spectrophotometry (ND-1000, NanoDrop Technologies, Wilmington, DE). The expression level was evaluated on an oligo nucleotide microarray (Whole Rat Genome microarray 22 K, Agilent Technologies, Santa Clara, CA) in 1) CP and WKY at 6 weeks of age, 2) CP and Lean at 6 weeks of age, 3) CP and WKY at 25 weeks of age, and 4) CP and Lean at 25 weeks of age. Two microarray slides were used for each comparison between two groups in two different RNA samples from three different rats per group.

### Hybridization, microarray scanning, and data analysis

The reverse transcription labeling and hybridization were conducted using the Agilent 60-mer Oligo microarray (Agilent Technologies), according to the instructions provided by the manufacturer. After hybridization, the glass slides were scanned with an Agilent DNA MicroArray Scanner (Agilent Technologies) containing a 532 nm laser for Cy3 measurement and a 635 nm laser for Cy5 measurement. Microarray data analysis was carried out using GeneSpring ver. 7.3.1 software (Agilent Technology) [18]. The detected signals were subjected to normalization using GeneSpring normalization algorithms. In each comparison, genes were filtered based on minimum ±1.0 of log ratio (WKY vs. CP or Lean vs. CP) and P < 0.01 using the Student’s *t*-test. Gene Ontology (GO) analysis was performed using GeneSpring (Agilent Technology).

### Protein samples preparation and separation by two-dimensional fluorescence difference gel electrophoresis (2D-DIGE) and data analysis

Frozen tissue sections were homogenized in lysis buffer (30 mM Tris–HCl, 7 M urea, 2 M thiourea, 4% w/v CHAPS, and a protease inhibitor cocktail, pH 8.5). Protein extracts were labeled with either Cy3 or Cy5 for comparison on the same two-dimensional (2D) gel. The epididymal adipose tissue mixture was labeled with Cy2 for the use as an internal standard for every gel electrophoresis and 2DE was performed as described previously [19]. Images were visualized using Typhoon 9400 fluorescence scanner (GE Healthcare, Buckinghamshire, UK) at 488/520 nm for Cy2, 532/580 nm for Cy3 and 633/670 nm for Cy5 dyes. The differential in-gel analysis module of the DeCyder software (GE Healthcare) was used for automatic detection followed by editing of protein spots. The same software was used for abundance measurements for each gel by comparing normalized volume ratios of individual spots from Cy3- or Cy5-labeled samples to corresponding Cy2-signals from pooled samples (internal standard) [15]. Thereafter, all gel comparisons and initial screening type statistical analyses were performed with the biological variation analysis module. Two-tailed Student’s *t*-test was used to determine differences between paired groups (WKY vs. CP or Lean vs. CP) using this module.

### Protein identification

After image analysis, gels containing the additional load of unlabeled proteins from the epididymal adipose tissue were stained with Colloidal Coomassie Brilliant Blue G (GE Healthcare) and matched to the fluorescent 2D-DIGE images. Selected spots were picked and in-gel digestion of protein samples was performed using the protocol described previously by our group [15]. The peptide mixtures were analyzed using matrix-assisted laser desorption ionization time-of-flight tandem mass spectrometry (MALDI-TOF/TOF MS; 4800 *Plus* MALDI TOF/TOF™ Analyzer, Applied Biosystems, Foster City, CA) operating in positive-ion reflector mode. Protein database search was performed with the Paragon Method using Protein Pilot software (Applied Biosystems) to identify excised proteins.

### Quantitative RT-PCR

To confirm the gene expression of identified proteins, total RNA extracted from the adipose tissue (n = 6 in each group) was subjected to quantitative reverse transcription and polymerase chain reaction (RT-PCR) analysis with primers specific for mRNAs encoding serine protease inhibitor A3L (*Spin2b*), alpha-2-HS-glycoprotein (*Ahsg*), carboxylesterase 3 (*Ces3*), monoglyceride lipase (*Mgll*), and pyruvate dehydrogenase E1 component subunit beta (*Pdhd1*), using ABI PRISM 7000 Sequence Detection System (Applied Biosystems). All primers and FastStart Universal Probes were designed from the Assay Design Center/Probe Finder (Roche Applied Science, Indianapolis, IN). Standard curves were generated for each primer set and a coefficient file generated to quantify the expression of each gene relative to β-actin. All experiments were performed in duplicates.

### Western blot analysis

To confirm the results of proteomic analysis, western blot analysis was conducted. For this purpose, samples (n = 4 or 6 in each group) were separated by 12% SDS-PAGE and electroblotted onto polyvinylidenedifluoride (PVDF) membranes. The membranes were incubated with goat polyclonal antibodies to rat AHSG (Santa Cruz Biotechnology, Santa Cruz, CA) at a 1:10,000 dilution and to human CES3 (Santa Cruz Biotechnology) at a 1:10,000 dilution, and a rabbit polyclonal antibody to MGLL (Abcam, Cambridge, UK) at a 1:5,000 dilution. Mouse anti-β-actin (ACTB) monoclonal antibody (Sigma, St Louis, MO) at a 1:5,000 dilution was used as a loading control. The protein bands were visualized by ECL plus Western blotting detection system (GE Healthcare) and Quantity One v3.0 software (Bio-rad Laboratories) was used to quantitate the band intensities. Protein expression levels were normalized relative to the level of β-actin protein in the same tissue sample.

### Statistical analysis

Data are presented as mean ± SEM. Regarding the physiological and biochemical parameters and quantitated data by both RT-PCR and western blot analyses, statistical significance was evaluated by one-way analysis of variance followed by Dunnett’s post hoc test using the JMP 8.0 software (SAS Institute Inc., Cary, NC). A P value <0.05 was considered statistically significant.

## Results

### Body weight, epididymal adipose tissue weight, systolic blood pressure, and plasma biochemical data

**Figure 1:**
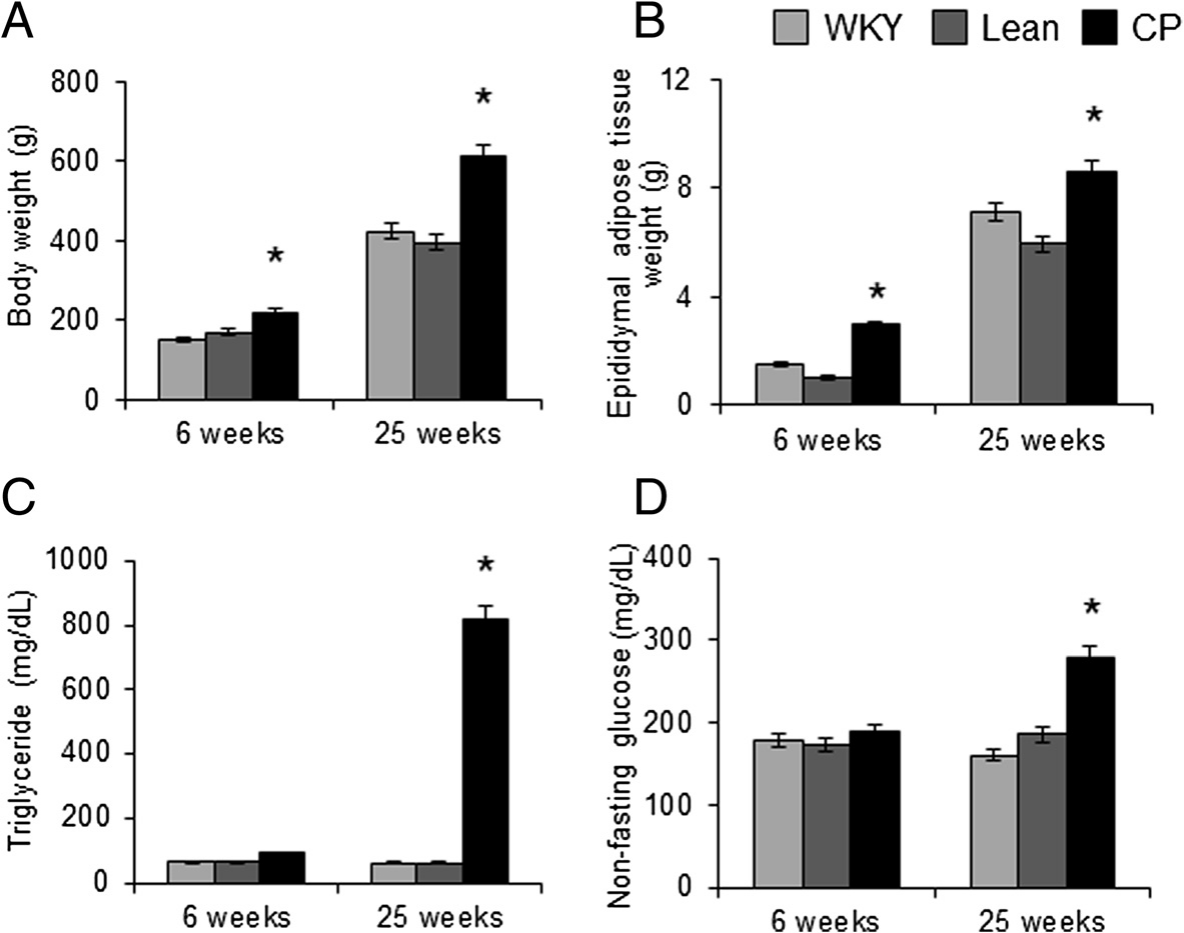
**Body weight and plasma biochemical data. (A)** Body weight, **(B)** epididymal adipose tissue weight, and plasma levels of **(C)** triglyceride and **(D)** non-fasting glucose in WKY, Lean, and CP rats at 6 and 25 weeks of age. Data are mean ± SEM of six animals per group. *P < 0.05, versus the corresponding value for WKY. WKY; Wistar Kyoto rats, Lean; spontaneously hypertensive rats (SHR/lean), CP; SHR/NDmcr-cp (cp/cp) rats.

Body weight and epididymal adipose tissue weight of 6- and 25-week-old CP rats were significantly greater than age-matched WKY and Lean rats (Figure 1A, B). There were no significant differences between WKY and Lean rats at both 6 and 25 weeks of age. Hypertension was observed in both CP and Lean rats aged 6 and 25 weeks, and there was no significant difference in systolic blood pressure between the two strains (data not shown). At 6 weeks of age, there were no significant differences in triglyceride and non-fasting glucose levels among the three groups (Figure 1C, D). At 25 weeks of age, the plasma levels of triglyceride and non-fasting glucose were significantly higher in CP than in WKY and Lean rats (Figure 1C, D).

### Microarray analysis of gene expression profiles

After the experiments of microarray analysis, all hybridization spots on the image were quantified. The intensity of fluorescence of data was converted into Log_10_ values and analyzed. After excluding data with *P* values of >0.01, we analyzed the extracted data and obtained the genes that showed significant differences between CP and WKY or Lean rats. On the oligo DNA microarray, 535 or 318 genes were significantly up-regulated (*p* < 0.01) in 6-week-old CP compared with age-matched WKY and Lean rats, respectively. Furthermore, 508 and 446 genes were down-regulated in 6 weeks-old CP compared with WKY or Lean rats of the same age, respectively. In 25-week-old rats, significant up-regulation of 277 and 286 genes, and down-regulation of 224 and 638 genes were noted in CP compared with WKY and Lean rats, respectively. Moreover, 33 genes were significantly up-regulated and 17 genes were significantly down-regulated in the epididymal adipose tissues of CP compared with WKY and Lean rats at both 6 and 25 weeks of age (Additional file 1: Tables S1 and S2). To understand their biological roles, the genes with significantly different expression levels by microarray analysis were assigned to established GO classification by GeneSpring ver.7.3.1. In GO classification, three aberrant GO terms (*p <* 0.01) were found in up-regulated genes in CP compared with WKY and Lean rats at both 6 and 25 weeks of age; transferases, electron transport, and electron transporter activity (Table 1). Based on the Kyoto Encyclopedia of Genes and Genomes (KEGG) molecular pathway analysis, genes with significantly different expression levels by microarray analysis were also assigned to KEGG molecular pathway by GeneSpring. The analysis identified 5 predicted pathways for the up-regulated genes in CP compared with WKY and Lean rats at both 6 and 25 weeks of age. These included peroxisome proliferator-activated receptor (PPAR) signaling pathway, carbon fixation, pentose phosphate pathways, glutathione metabolism, and alkaloid biosynthesis I (Table 1).

**Table 1:** List of entities identified by GO and the pathways identified by KEGG of significantly up-regulated and down-regulated genes in adipose tissues of 6- and 25-week-old CP rats

**GO accession**	**GO term**	***P *** **value**			
		**6-week-old**		**25-week-old**	
		**WKY-CP**	**Lean-CP**	**WKY-CP**	**Lean-CP**
*Up-regulation*					
GO:0016740	Transferase	1.4E-14	2.0E-08	6.8E-06	4.4E-06
GO:0006118	Electron transport	8.0E-08	4.1E-03	3.6E-03	1.7E-04
GO:0005489	Electron transporter activity	2.6E-07	1.0E-02	3.7E-03	1.6E-04
*Down-regulation*					
GO:0016740	Transferase	5.43E-02	3.49E-03	2.68E-04	1.41E-11
GO:0005489	Electron transporter activity	1.09E-01	1.26E-03	1.71E-02	1.80E-05
GO:0006118	Electron transport	1.11E-01	1.30E-03	1.73E-02	1.87E-05
Predicted pathway					
*Up-regulation*					
PPAR signaling pathway		4.25E-11	2.71E-03	9.73E-03	5.54E-02
Carbon fixation		4.50E-07	1.10E-05	2.33E-03	3.11E-02
Pentose phosphate pathway		2.84E-05	1.45E-03	3.92E-05	1.90E-01
Glutathione metabolism		2.14E-04	4.55E-03	1.95E-04	2.21E-04
Alkaloid biosynthesis I		9.47E-03	1.00E + 00	2.62E-03	2.79E-03
*Down-regulation*					
ECM-receptor interaction		7.40E-04	1.64E-02	1.09E-02	3.85E-03
Glycolysis _ Gluconeogenesis		1.59E-03	4.60E-04	5.56E-02	1.42E-01
Fructose and mannose metabolism		3.09E-03	1.12E-03	3.01E-02	3.80E-01
Cell Communication		9.95E-03	2.64E-03	4.71E-02	5.18E-02
Carbon fixation		8.65E-02	4.63E-04	1.27E-03	4.82E-04

WKY; Wistar Kyoto rats, Lean; spontaneously hypertensive rats (SHR/lean), CP; SHR/NDmcr-cp (cp/cp) rats.

### Comparison of protein identification

**Figure 2:**
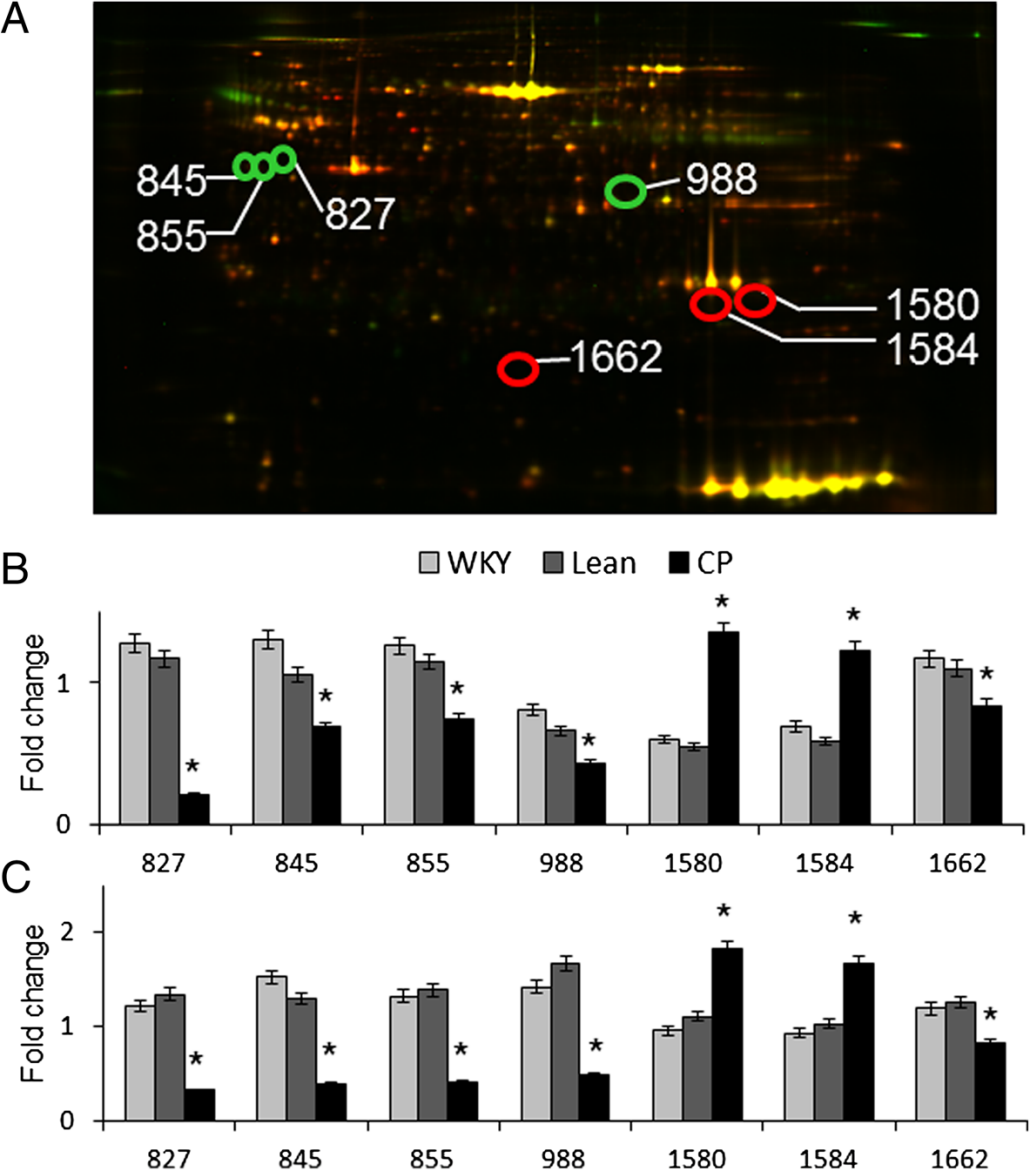
**Representative 2D-DIGE image of adipose lysates from 25-week-old Lean and CP rats. (A)** The proteins (40 μg each) were labeled with Cy3 and Cy5 dyes, mixed and subjected to 2D-DIGE analysis. We selected significantly different spots of 6- and 25-week-old CP compared with age-matched control (WKY and Lean) rats. The expression level was quantified in each strain at 6 **(B)** and 25 weeks of age **(C)** and expressed as mean ± SEM of four rats per group. *P < 0.05, versus the corresponding value for WKY or Lean rats. WKY; Wistar Kyoto rats, Lean; spontaneously hypertensive rats (SHR/lean), CP; SHR/NDmcr-cp (cp/cp) rats.

To investigate differences in protein expression in the adipose tissues, epididymal adipose tissue proteins from 6- and 25-week-old WKY, Lean, and CP rats were extracted and subjected to comparative analysis by DIGE. Typical gels are shown in Figure 2A. Seven spots in the 2-DE gels were significantly different in 6- and 25-week-old CP, WKY and Lean rats. These spots were isolated and subjected to MALDI-TOF-MS. The peptides mass peaks were compared with those in the NCBI database and the proteins of the seven spots were identified (Table 2). The expression levels of proteins of spots 1580 and 1584 were significantly up-regulated while those of spots 827, 845, 855, 988, and 1662 were significantly down-regulated in CP compared with WKY and Lean rats at both 6 (Figure 2B) and 25 weeks of age (Figure 2C). The identified proteins were serine protease inhibitor A3L (Spin2b) (spot 827), alpha-2-HS-glycoprotein (AHSG) (spots 845 and 855), carboxylesterase 3 (CES3) (spot 988), monoglyceridelipase (MGLL) (spots 1580 and 1584), and pyruvate dehydrogenase E1 component subunit beta (PDHB1) (spot 1662).

**Table 2:** List of proteins with significant differences in their levels in epididymal adipose tissues of 6- and 25-week-old CP, WKY, and Lean rats

**Spot**	**Protein name**	**% cov.**	**Peptides (95%)**	**Fold change up(+)/down(-)**			
				**6-week-old**		**25-week old**	
								**WKY-CP**	**Lean-CP**	**WKY-CP**	**Lean-CP**
827	Serine protease inhibitor A3L	12.1	4	-1.74	-1.6	-3.74	-4.12
845	Alpha-2-HS-glycoprotein	10.5	3	-1.9	-1.17	-3.98	-3.4
855	Alpha-2-HS-glycoprotein	10.5	3	-1.71	-1.56	-3.17	-3.33
988	Carboxylesterase 3	11	5	-1.89	-1.53	-2.9	-3.4
1580	Monoglyceride lipase	21.8	6	2.26	2.45	1.89	1.64
1584	Monoglyceride lipase	20	2	1.77	2.08	1.79	1.63
1662	Pyruvate dehydrogenase E1 component subunit β	31.8	5	-1.39	-1.31	-1.44	-1.53

Abbreviations as in Table 1.

### Expression of identified proteins

**Figure 3:**
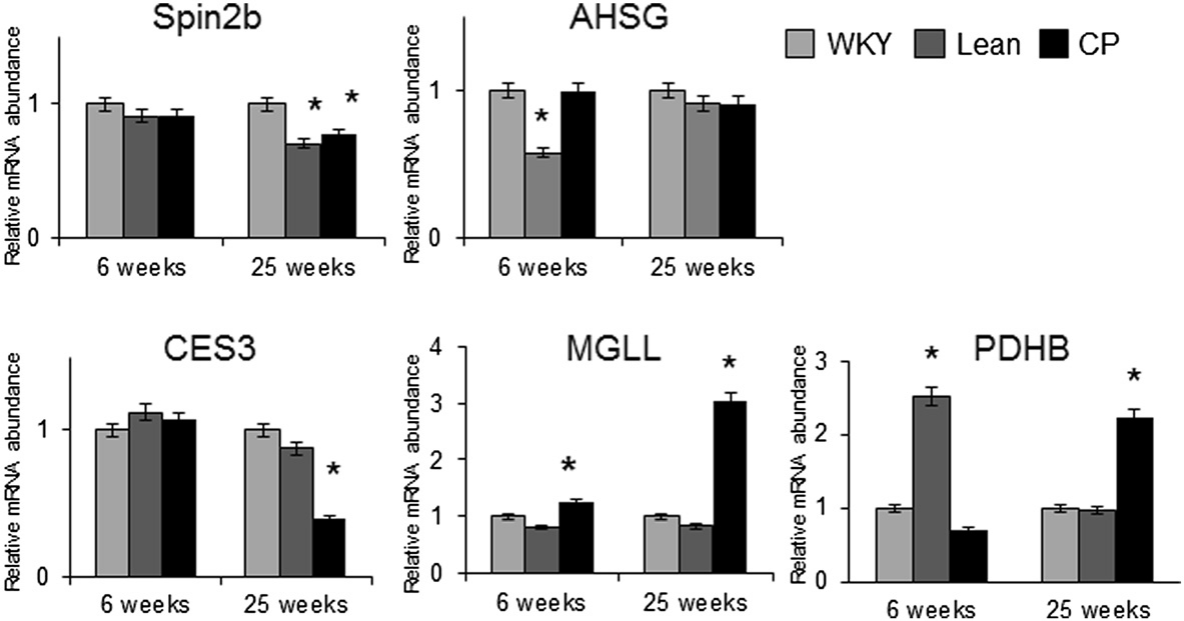
**Gene expression of the identified proteins in WKY, Lean, and CP rats at 6 and 25 weeks of age.** The mRNA levels of Spin2b, AHSG, CES3, MGLL, and PDHB1 in the adipose tissues were determined by quantitative RT-PCR analysis and expressed relative to that of β-actin. Quantitative data are expressed relative to the values for WKY. Data are mean ± SEM of six animals per group. *P < 0.05, versus the corresponding value for WKY. WKY; Wistar Kyoto rats, Lean; spontaneously hypertensive rats (SHR/lean), CP; SHR/NDmcr-cp (cp/cp) rats.

**Figure 4:**
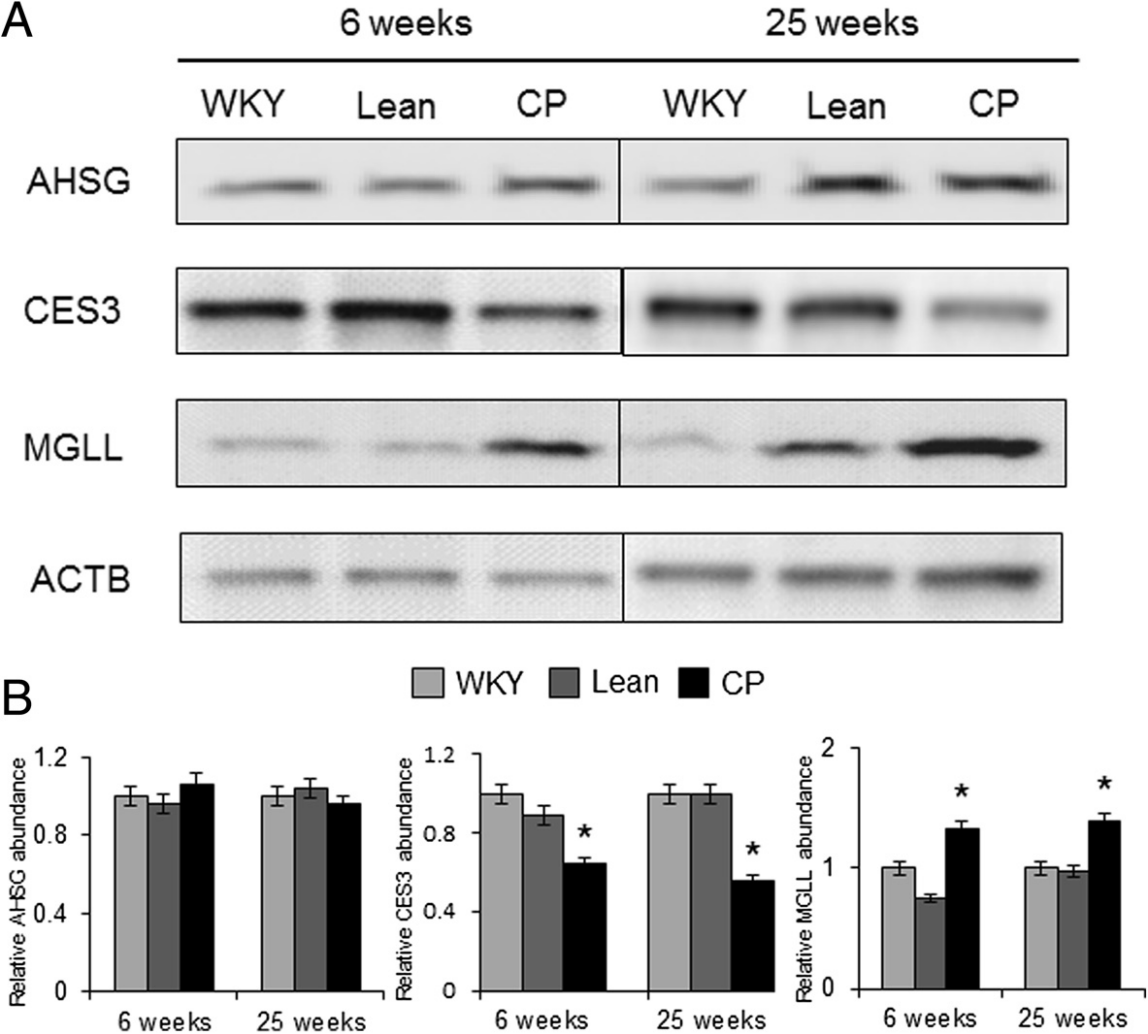
**The amounts of AHSG, CES3, and MGLL in WKY, Lean, and CP rats at 6 and 25 weeks of age. (A)** Representative immunoblots of each protein. **(B)** The amount of each protein quantitated relative to the amount of β-actin and expressed relative to the value of WKY. Data are mean ± SEM of six animals per group. *P < 0.05, versus the corresponding value for WKY. WKY; Wistar Kyoto rats, Lean; spontaneously hypertensive rats (SHR/lean), CP; SHR/NDmcr-cp (cp/cp) rats.

The gene expression levels of the five identified proteins were confirmed by quantitative RT-PCR analysis. At 25 weeks of age, the mRNA levels of *Mgll* and *Pdhb1* were significantly greater while those of *Spin2b* and *CES3* were significantly lower in CP compared with age-matched WKY rats (Figure 3). Western blot analysis was also performed to confirm the results of proteomics analysis. The expression of MGLL was significantly up-regulated while that of CES3 was significantly down-regulated in 6-and 25-week-old CP compared with age-matched control (WKY and Lean rats) (Figure 4). These findings were consistent with those of proteomic analysis.

## Discussion

In the present study, we performed genomic and proteomic analyses to identify the target molecules associated with obesity related to the metabolic syndrome. More than one thousand genes were significantly different in the microarray analysis and many hundred proteins were significantly different in the proteomic analysis among the three rat groups at 6 or 25 weeks of age. Among them, microarray analysis identified 33 significantly up-regulated genes and 17 significantly down-regulated genes in adipose tissues of 6- and 25-week-old CP, compared with age-matched WKY and Lean rats. Proteomics analysis identified significantly different five proteins in CP than WKY and Lean rats at both 6 and 25 weeks of age. Only a few of the genes showed similar changes both in the microarray and the proteomics analysis. Previous studies using transcriptomic and proteomics analyses demonstrated a positive correlation between transcript and protein levels for the majority of molecules [20], but other studies reported limited correlation between transcription and translation in mammals [21]. The different expression patterns noted in the two methodological approaches might arise from limitation of detection sensitivity or electrophoretic separation. Moreover, the discrepancy between the microarray and proteomics analyses might be due to differential regulation of translation, turnover, or alternative splicing.

With the gene-by-gene approach, it would be difficult and time-consuming to obtain a global picture of gene expression patterns. The cDNA and oligo array technology allows simultaneous comparison of thousands of mRNAs from a given tissue, and thus provides a comprehensive assessment of expression levels. So far, microarray analysis is a powerful strategy for research in the obesity field [22,23]. In the present study, we applied comparative genomic and proteomic analyses of adipose tissue to identify the molecular targets associated with obesity related to the metabolic syndrome. Microarray analysis identified 33 significantly up-regulated genes and 17 significantly down-regulated genes in the epididymal adipose tissues of CP compared with WKY and Lean rats at both 6 and 25 weeks of age. Among them, only CES3 showed significant differences in expression in both 6- and 25-week-old rats by proteomics analysis. CES3, also called triacylglycerol hydrolase, is an endoplasmic reticulum lipase which belongs to the type-B carboxylesterase/lipase family [24]. These carboxylesterases are responsible for the hydrolysis of ester- and amide-bond-containing drugs, such as cocaine and heroin, and are involved in the detoxification of xenobiotics and activation of ester and amide prodrugs [25]. CES3 is highly expressed in the liver, adipose tissue and also expressed in the small intestine, heart, and kidney [26]. The specific function of this enzyme has not yet been determined; however, it is speculated that carboxylesterases may play a role in lipid metabolism because endobiotics, such as triglyceride, cholesterol esters, and 2-arachidonoylglycerol are also substrates for carboxylesterases [27,28]. Given that carboxylesterases hydrolyzes long-chain fatty acid esters and thioesters, these enzymes have been considered therapeutic targets in the treatment of metabolic disorders, such as diabetes and atherosclerosis [29]. In the present study, the expression of CES3 was significantly down-regulated in the adipose tissue of CP compared with WKY and Lean rats at the two tested ages. These results suggest that a decrease in lipolytic enzyme activity could affect the hydrolysis of triglyceride to fatty acids and glycerol, leading to its accumulation in adipose tissue. In a study using CES3 knockout mice, global deletion of CES3 resulted in significant decreases in plasma triglyceride and fatty acid levels although the weight of white adipose tissue was greater in CES3 knockout mice compared with wild-type mice [30]. Moreover, CES3 knockout mice showed the improvement of insulin sensitivity and glucose tolerance. Since CP rats were hyperglycemic and hyperinsulinemic, the lipid profiles in our CP rats are different from those of the CES3 knockout mice. Taken together with the results of these previous studies, CES3 might be down-regulated in CP rats as a result of hyperglycemia or physiological adaptation to obesity.

In the present study, MGLL was up-regulated in adipose tissues of CP compared with age-matched controls. Adipose tissue triglycerides are likely hydrolyzed in a three-step process catalyzed by adipose triglyceride lipase, hormonesensitive lipase, and MGLL [31]. MGLL is a glyceride consisting of one fatty acid chain covalently bonded to a glycerol molecule through an ester linkage [32]. MGLL hydrolyzes monoglyceride to free glycerol and fatty acid, and is considered the rate-limiting step of monoglyceride hydrolysis in mammals [33]. A previous study showed that whole-body MGLL knockout mice exhibited decreased forskolin-stimulated fatty acid and glycerol release from white adipose tissues and attenuated high-fat diet-induced insulin resistance [34]. Given that augmented fatty acid release in the basal condition caused by increased lipolytic enzymes is implicated in the development of insulin resistance of adipocytes, up-regulation of MGLL in CP rats may contribute to the pathogenesis or progression of obesity related to the metabolic syndrome. It is known that perilipins (e.g. perilipin 1) and other lipases, such as hormone-sensitive lipase and adipose triglyceride lipase are also pivotal in the regulation of adipose tissue fat metabolism. However, there were no significant differences in the expression of these lipases, between CP and control (WKY and Lean rats) at 6 or 25 weeks of age.

In the present microarray analysis, KEGG annotation revealed significant differences in proteins associated with the PPAR signaling pathway. PPARs are ligand-activated transcription factors belonging to the nuclear receptor superfamily of regulatory factors [35]. They exercise homeostatic functions in the intestine at the interface between nutrient metabolism and immunity, and their main function is related to the regulation of genes involved in glucose and lipid metabolism [36,37]. PPAR family is also involved in the regulation of inflammation and energy homeostasis [38]. Among them, PPARα directly governs the triglyceride hydrolysis pathway in the liver [39]. CES3 and MGLL are induced in mouse hepatocytes by short-term treatment with PPARα agonist [40], but the present result showed down-regulation of CES3 in spite of up-regulation of PPAR signaling pathway. Further studies are needed to clarify the relationship between CES3 and PPAR signaling, but the results suggest that PPAR signaling could be important targets in obesity, obesity-induced inflammation, and metabolic syndrome.

In the present study, we applied comparative genomic and proteomic analyses in adipose tissues to identify the molecular targets associated with obesity related to the metabolic syndrome. Microarray analysis identified 33 significantly up-regulated genes and 17 significantly down-regulated genes in the adipose tissues of 6- and 25-week-old CP, compared with age-matched WKY and Lean rats. Based on the classification of genes with significant difference in expression according to their function, a large proportion of the identified genes are known to be involved in transferase, electron transport, and electron transporter activity. In KEGG molecular pathway analysis, the up-regulated genes were involved in PPAR signaling pathway. The up-regulated proteins identified by proteomics analysis and confirmed by western blot analysis, CES3 and MGLL were involved in adipose lipolysis and regulated PPAR signaling. These findings suggest that the proteins associated with adipocyte lipolysis and PPAR signaling may be involved in obesity related to the metabolic syndrome.

## Abbreviations

2D-DIGE: Two-dimensional fluorescence Difference gel electrophoresis; ACTB: β-actin; AHSG: Alpha-2-HS-glycoprotein; CES3: Carboxylesterase 3; CP: Spontaneously hypertensive rats/NDmcr-cp; GO: Gene Ontology; KEGG: Kyoto Encyclopedia of Genes and Genomes; Lean: Spontaneously hypertensive rats/lean; MALDI-TOF/TOF MS: Matrix-assisted laser desorption ionization time-of-flight tandem mass spectrometry; MGLL: Monoglyceride lipase; PDHB1: Pyruvate dehydrogenase E1 component subunit beta; PPAR: Peroxisome proliferator-activated receptor; PVDF: Polyvinylidenedifluoride; RT-PCR: Quantitative reverse transcription and polymerase chain reaction; SEM: Standard error; SHR: Spontaneously hypertensive rats; Spin2b: Serine protease inhibitor A3L; WKY: Wistar Kyoto rats.

## Competing interests

The authors declare no conflict of interest.

## Authors’ contributions

JC performed the experiments, analysis, and manuscript writing; SO contributed to study design and performed the proteomics analysis; HI, EK, IT, and MY participated in data interpretation of microarray analysis; CA performed the microarray analysis; YY participated in data interpretation of proteomics analysis; GI and MK contributed to study design and manuscript writing; SI participated in organization of the study, data interpretation, and preparation of the manuscript. All authors read and approved the final manuscript.

## Supplementary Material

Additional file 1 **DNA microarray results. Table S1.** Significantly up-regulated genes in CP rats compared with WKY and Lean rats at 6 and 25 weeks of age. **Table S2.** Significantly down-regulated genes in CP rats compared with WKY and Lean rats at 6 and 25 weeks of age. Click here for file
