# Supplementary material for: Expression of proteins associated with adipocyte lipolysis was significantly changed in the adipose tissues of the obese spontaneously hypertensive/NDmcr-cp rat
Source: Diabetol Metab Syndr. 2014 Jan 27;6:8. doi: 10.1186/1758-5996-6-8 (PMC3937142; doi:10.1186/1758-5996-6-8)
Supplement: Additional file 1 — DNA microarray results. Table S1. Significantly up-regulated genes in CP rats compared with WKY and Lean rats at 6 and 25 weeks of age. Table S2. Significantly down-regulated genes in CP rats compared with WKY and Lean rats at 6 and 25 weeks of age. [file 1758-5996-6-8-S1.docx]

**Additional file 1: DNA microarray results.**

**Table S1.** Significantly up-regulated genes in CP rats compared with WKY and Lean rats at 6 and 25 weeks of age.

| Gene name | Symbol | Log ratio | | | |
| --- | --- | --- | --- | --- | --- |
|  |  | 6-week-old | | 25-week-old | |
|  |  | WKY-CP | Lean-CP | WKY-CP | Lean-CP |
| acyl-CoA synthetase long-chain family member 4 | Acsl4 | 0.474 | 0.305 | 0.293 | 0.296 |
| adipose differentiation-related protein | ADRP | 0.738 | 0.604 | 0.811 | 0.904 |
| ATPase, Na+/K+ transporting, alpha 3 polypeptide | Atp1a3 | 0.666 | 0.737 | 0.156 | 0.172 |
| cDNA clone RGIGO67 5' end | AW919929 | 0.313 | 0.363 | 0.139 | 0.150 |
| cDNA clone RGIGU75 5' end | AW920373 | 0.654 | 0.478 | 0.282 | 0.185 |
| cDNA clone MGC:95070 IMAGE:7125327 | BC083855 | 0.540 | 0.282 | 0.233 | 0.331 |
| cDNA clone nrpc1-00003-h8 5' | CB546750 | 0.257 | 0.158 | 0.151 | 0.181 |
| similar to Integrin beta-2 precursor | Cd18 | 0.743 | 0.703 | 0.417 | 0.363 |
| CD44 antigen | Cd44 | 0.475 | 0.348 | 0.224 | 0.159 |
| cytochrome P450, subfamily 51 | Cyp51 | 0.464 | 0.299 | 0.303 | 0.257 |
| similar to RIKEN cDNA 3110037K17 | Dcir3 | 0.210 | 0.309 | 0.220 | 0.197 |
| FXYD domain-containing ion transport regulator 6 | Fxyd6 | 0.222 | 0.188 | 0.681 | 0.603 |
| gamma-glutamyl hydrolase | Ggh | 0.740 | 0.350 | 0.296 | 0.505 |
| glutaredoxin 1 (thioltransferase) | Glrx1 | 0.306 | 0.171 | 0.436 | 0.379 |
| glycoprotein (transmembrane) nmb | Gpnmb | 0.862 | 0.982 | 0.405 | 0.364 |
| immunoglobulin superfamily, member 6 | Igsf6 | 0.362 | 0.379 | 0.261 | 0.134 |
| hypothetical LOC299269 | isg12(b) | 0.658 | 0.394 | 0.388 | 0.247 |
| potassium intermediate/small conductance calcium-activated channel, subfamily N, member 4 | Kcnn4 | 0.593 | 0.536 | 0.287 | 0.338 |
| lysosomal-associated protein transmembrane 5 | Laptm5 | 0.361 | 0.488 | 0.752 | 0.910 |
| lipase A, lysosomal acid | Lipa | 0.330 | 0.463 | 0.346 | 0.288 |
| similar to MD-1 | LOC291359 | 0.260 | 0.249 | 0.226 | 0.194 |
| similar to high affinity immunoglobulin gamma Fc receptor I | LOC295279 | 0.318 | 0.418 | 0.182 | 0.230 |
| similar to GLI pathogenesis-related 1 (glioma); related to testes-specific, vespid, and pathogenesis proteins | LOC299783 | 0.418 | 0.320 | 0.360 | 0.236 |
| similar to triggering receptor TREM-2A | LOC301227 | 0.425 | 0.732 | 0.190 | 0.453 |
| similar to putative nuclear protein | LOC301570 | 0.520 | 0.302 | 0.294 | 0.257 |
| similar to coactosin-like 1; coactosin-like protein | LOC361422 | 0.214 | 0.270 | 0.347 | 0.623 |
| lysozyme | Lyz | 0.554 | 0.547 | 0.966 | 0.971 |
| protein kinase C, delta | Prkcd | 0.331 | 0.274 | 0.142 | 0.158 |
| protein tyrosine phosphatase, non-receptor type substrate 1 | Ptpns1 | 0.239 | 0.231 | 0.374 | 0.226 |
| syndecan 1 | Sdc1 | 0.366 | 0.244 | 0.300 | 0.358 |
| secreted phosphoprotein 1 | Spp1 | 1.749 | 1.730 | 1.011 | 0.489 |
| thromboxane A synthase 1 | Tbxas1 | 0.345 | 0.391 | 0.196 | 0.184 |
| BC003208 Col17a1 protein | TC472619 | 0.495 | 0.410 | 0.139 | 0.279 |

WKY; Wistar Kyoto rats, Lean; spontaneously hypertensive rats (SHR/lean), CP; SHR/NDmcr-cp (cp/cp) rats.

**Table S2.** Significantly down-regulated genes in CP rats compared with WKY and Lean rats at 6 and 25 weeks of age.

| Gene name | Symbol | Log ratio | | | |
| --- | --- | --- | --- | --- | --- |
|  |  | 6-week-old | | 25-week-old | |
|  |  | WKY-CP | Lean-CP | WKY-CP | Lean-CP |
| adipsin | Adn | -0.479 | -0.395 | -0.681 | -0.590 |
| adrenergic receptor, beta 3 | Adrb3 | -0.428 | -0.332 | -0.201 | -0.843 |
| cDNA clone RGIBW93 5' end similar to laminin γ1 | AW141503 | -0.177 | -0.194 | -0.264 | -0.213 |
| cDNA clone RGIGT40 5' end | AW920282 | -0.559 | -0.347 | -0.826 | -0.220 |
| cDNA clone UI-R-BS2-bey-b-07-0-UI 3' | BF398315 | -0.333 | -0.518 | -0.527 | -0.421 |
| carboxylesterase 3 | Ces3 | -0.301 | -0.271 | -0.507 | -0.480 |
| dipeptidase 1 | Dpep1 | -0.361 | -0.329 | -0.267 | -0.150 |
| insulin-like growth factor 1 | Igf1 | -0.218 | -0.293 | -0.504 | -0.425 |
| scf mRNA for stem cell factor KL-2 | Kitl | -0.246 | -0.182 | -0.198 | -0.203 |
| similar to laminin-2 alpha2 chain precursor | LOC309368 | -0.287 | -0.194 | -0.218 | -0.131 |
| latent transforming growth factor beta binding protein 1 | Ltbp1 | -0.231 | -0.298 | -0.453 | -0.189 |
| mesothelin | Msln | -0.577 | -0.296 | -0.312 | -0.484 |
| regulator of G-protein signalling 3 | Rgs3 | -0.416 | -0.163 | -0.146 | -0.219 |
| solute carrier family 22, member 3 | Slc22a3 | -0.179 | -0.541 | -0.199 | -0.913 |
| C561_MOUSE Cytochrome b561 | TC470526 | -0.338 | -0.141 | -0.738 | -0.690 |
| tumor protein, translationally-controlled 1 | Tpt1 | -0.202 | -0.247 | -0.236 | -0.187 |
| vitamin K epoxide reductase complex, subunit 1 | Vkorc1 | -0.269 | -0.220 | -0.232 | -0.192 |

Abbreviations as in Additional file 1.
